# An Uncommon Cause of Acute Pancreatitis in a Patient With COVID-19

**DOI:** 10.7759/cureus.27910

**Published:** 2022-08-12

**Authors:** Francisco Vara-Luiz, Fábio Pé D’Arca Barbosa, Ana Antunes Albuquerque, Ana Valada Marques, Vanda Spencer

**Affiliations:** 1 Gastroenterology, Hospital Garcia de Orta, Almada, PRT; 2 Internal Medicine, Hospital Garcia de Orta, Almada, PRT

**Keywords:** covid-19, abdominal pain, dexamethasone, steroid-induced pancreatitis, drug-induced pancreatitis

## Abstract

Drug-induced pancreatitis is a rare though important condition that remains a diagnostic challenge. Most of the evidence relies on case reports, and clinicians should consider a high suspicion of the diagnosis after ruling out other causes. In particular, steroids are frequently used drugs that have recently been associated with acute pancreatitis. The authors present the case of a 60-year-old female admitted to the emergency room with a fever and shortness of breath. The SARS-CoV-2 test was positive, and the chest radiography was suggestive of COVID-19 pneumonia. The patient started dexamethasone because of respiratory failure. On Day 7, she developed epigastric pain radiating to the back and the amylase level was greater than 10 times the upper reference limit (1354 U/L). A detailed evaluation of the medical history, along with the exclusion of other possible etiologies confirmed the diagnosis of steroid-induced pancreatitis. Supportive care and cessation of the offending drug led to the resolution of symptoms. As steroids are used as part of the treatment of most COVID-19 patients, this case suggests the need to consider this entity, as a delay in the diagnosis may result in complications and prolonged hospital stay.

## Introduction

Acute pancreatitis (AP) is the most common pancreatic condition, being the gastrointestinal disease most frequently requiring acute hospitalization [1]. Several conditions are associated with AP, with gallstones, alcohol use and hypertriglyceridemia being the most frequent. AP due to medications is a rare condition (1-4%) [2] which tends to occur within the first four to eight weeks after starting a drug. Steroid-induced pancreatitis has been reported, particularly four to 14 days after drug initiation, and the mechanism seems to result from the increased viscosity of the pancreatic juice [3]. Proper diagnosis of the condition is important as a delay may result in extended hospital stays, increased morbidity, and healthcare costs [4]. We report a case of steroid-induced pancreatitis in a patient with COVID-19, a viral infection recently associated with AP [5].

## Case presentation

A 60-year-old Caucasian female with no past medical history presented to the emergency room with complaints of progressively worsening fatigue, cough, myalgias, shortness of breath, and fever which started five days earlier. The chest radiography revealed bilateral airspace opacities and the SARS-CoV-2 real-time reverse transcriptase polymerase chain reaction test was positive. The patient was diagnosed with COVID-19 pneumonia and started oxygen supplementation (2 L/minute) and oral dexamethasone (6 mg) for respiratory failure (pO_2_55 mmHg; normal > 80 mmHg) that was improving during hospitalization. Symptomatic therapy with paracetamol was only needed on Day 1.

On Day 7, the patient started experiencing abdominal pain in the epigastric region with radiation to the back, intensity 9/10 and with no correlation to meals, as well as nausea. On physical examination, the patient was hemodynamically stable, and apyretic, with tenderness in the epigastric region. Blood analyses (Table 1) showed mild leukocytosis with neutrophilia and increased C-reactive protein. The amylase level was 1354 U/L (normal < 100 U/L). 

**Table 1:** Laboratory values. RBC: red blood cells; MCV: mean corpuscular volume; RDW: red cell distribution width; WBC: white blood cells; HDL: high-density lipoprotein; LDL: low-density lipoprotein; mg/dl: milligrams per deciliter; U/L: units per liter; g/dl: grams per deciliter; fL: femtoliter; mmol/L: millimoles per liter; µL: microliter.

Laboratory parameter	Patient values	Normal values
Complete blood count		
RBC (10^6^/µL)	4.25	3.63-5.04
Hemoglobin (g/dL)	12.5	12.0-15.3
Hematocrit (%)	37.5	34.7-45.1
MCV (fL)	88.4	80.0-100.0
RDW (%)	12	11.9-15.9
Platelets (10^3^/µL)	500	150-450
WBC	13.3	4.0-11.0
WBC Differential		
Neutrophil %	82	43.0-82.3
Lymphocyte %	12	14.5-45.2
Monocyte %	4	4.3-13.3
Eosinophil %	0.1	0.1-6.8
Basophil %	0	0.0-2.0
Chemistries		
Sodium (mmol/L)	138	135-145
Potassium (mmol/L)	4.1	3.5-5.0
Chloride (mmol/L)	99	95-108
Magnesium (mg/dL)	2.3	1.6-2.6
Blood urea nitrogen (mg/dL)	17	16-48
Creatinine (mg/dL)	0.9	0.5-0.9
Calcium (mg/dL)	8.5	8.1-10.2
Aspartate transaminase (U/L)	28	<32
Alanine transaminase (U/L)	45	<50
Alkaline phosphatase (U/L)	60	35-104
Total bilirubin (mg/dL)	0.4	<1.2
Albumin (g/dL)	3.8	3.5-5.0
Total protein (g/dL)	6.9	6.6-8.7
Amylase (U/L)	1354	<100
C-reactive protein (mg/dL)	20.69	<0.2
Total cholesterol (mg/dL)	130	<190
LDL cholesterol (mg/dL)	73	<135
HDL cholesterol (mg/dL)	45	>42
Triglycerides (mg/dL)	60	<150

**Figure 1:**
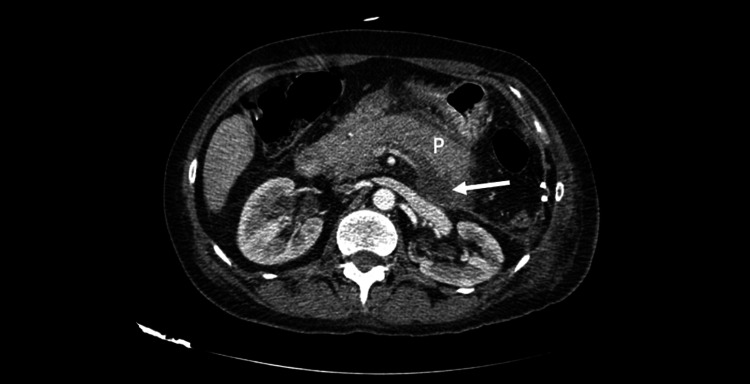
Abdominal computed tomography with contrast showing acute pancreatitis with diffuse swelling of the pancreas (P) and retropancreatic fluid collection with no signs of necrosis (arrow).

The diagnosis of AP was confirmed, and supportive measures (nothing by mouth, intravenous fluid therapy, pain control with paracetamol) were started. The abdominal ultrasound didn’t find suggestive findings of gallstone disease. An abdominal computed tomography scan (Figure 1) was performed due to the absence of improvement at 48 hours and showed a retropancreatic fluid collection, as well as no suggestive signs of mesenteric ischemia, a differential diagnosis of elevated amylase. Other common causes of pancreatitis were excluded: no gallstones could be identified, the patient denied alcohol use, and serum calcium, triglyceride, and hepatic panel were within the normal range, as depicted in Table 1. Autoimmune etiology was considered unlikely since the symptoms started during Day 7 of dexamethasone.

Steroid-induced pancreatitis was considered the most likely diagnosis based on the clinical history and laboratory findings. Dexamethasone was discontinued on Day 9 which was accompanied by clinical and analytical improvement. By the time of discharge, she was able to tolerate a soft diet with no abdominal pain, with instructions for outpatient follow-up with a gastroenterologist. The patient was advised to avoid steroid use. 

## Discussion

AP is an acute inflammatory process of the pancreas with variable involvement of other regional tissues or remote organ systems [6]. There is no specific drug to treat AP, and management includes supportive care along with treating the underlying cause and complications once they develop [7]. 

Drug-induced pancreatitis (DIP) is an uncommon and challenging diagnosis for clinicians that tends to be mild, self-limited, and with low mortality. DIP is rarely accompanied by clinical or laboratory evidence of a drug reaction such as eosinophilia or rash. More than 120 drugs have been implicated as etiologic agents of AP, with different pathophysiologic mechanisms according to the literature [8]. Although the World Health Organization has listed more than 500 drugs that could cause AP [9], only two have solid evidence from retrospective cohorts and randomized trials (azathioprine and didanosine), making the real magnitude of DIP still unknown [10]. As the current knowledge is very limited and the vast majority of the evidence is based on case reports, it is very important to rule out other etiologies before attributing a drug as the cause of AP. 

Badalov et al. [8] classified DIP into five classes based on the number of cases reported, consistent latency period, and reaction with rechallenge. Class I and II drugs have the greatest potential for causing AP and dexamethasone, the steroid used in this case, has been identified as a class IB drug. In contrast to other causes of DIP, it has been suggested that steroid-induced pancreatitis may be associated with a more severe course as a high proportion of case reports resulted in death [11].

AP has also been reported in COVID-19 infection, a potential confounder in this case, although the mechanism is still not very well understood. The association between AP and COVID-19 lacks further studies in order to investigate causality, as well as the severity of pancreatic inflammation and its correlation with the disease course [5]. Furthermore, COVID-19 as a cause of AP appears to be more frequent in those with critical illness [12], in contrast to this case, making dexamethasone, a steroid used to treat COVID-19 patients with oxygen requirements [13], the most likely cause of AP in this patient.

## Conclusions

This report is intended to alert physicians to the importance of timely recognition of DIP and provides further evidence of the relationship between dexamethasone and AP in the setting of COVID-19 infection. As steroids are used as part of the treatment of most COVID-19 patients, the clinician should maintain a high suspicion for this entity after other causes have been ruled out.
